# Association of preoperative albumin–bilirubin with surgical textbook outcomes following laparoscopic hepatectomy for hepatocellular carcinoma

**DOI:** 10.3389/fonc.2022.964614

**Published:** 2022-07-29

**Authors:** Fei-Qi Xu, Tai-Wei Ye, Dong-Dong Wang, Ya-Ming Xie, Kang-Jun Zhang, Jian Cheng, Zun-Qiang Xiao, Si-Yu Liu, Kai Jiang, Wei-Feng Yao, Guo-Liang Shen, Jun-Wei Liu, Cheng-Wu Zhang, Dong-Sheng Huang, Lei Liang

**Affiliations:** ^1^ General Surgery, Department of Hepatobiliary and Pancreatic Surgery and Minimal Invasive Surgery, Zhejiang Provincial People’s Hospital, Affiliated People’s Hospital, Hangzhou Medical College, Hangzhou, China; ^2^ The Second School of Clinical Medicine, Zhejiang Chinese Medical University, Hangzhou, China; ^3^ Department of Medical, Lishui Municipal Central Hospital, Lishui, China; ^4^ Department of Key Laboratory of Tumor Molecular Diagnosis and Individualized Medicine of Zhejiang Province, Zhejiang Provincial People's Hospital (People's Hospital of Hangzhou Medical College), Hangzhou, China; ^5^ School of Clinical Medicine, Hangzhou Medical College, Hangzhou, China

**Keywords:** hepatocellular carcinoma, hepatectomy, albumin–bilirubin, textbook outcomes, laparoscopic

## Abstract

**Background and aims:**

Recently, the effectiveness of “textbook outcomes (TO)” in the evaluation of surgical quality has been recognized by more and more scholars. This study tended to examine the association between preoperative albumin–bilirubin (ALBI) grades and the incidence of achieving or not achieving TO (non-TO) in patients with hepatocellular carcinoma (HCC) undergoing laparoscopic hepatectomy.

**Methods:**

The patients were stratified into two groups: ALBI grade 1 (ALBI ≤ -2.60) and ALBI grade 2/3 (ALBI > -2.60). The characteristics of patients and the incidence of non-TO were compared. Multivariate analyses were performed to determine whether ALBI grade was independently associated with TO.

**Results:**

In total, 378 patients were enrolled, including 194 patients (51.3%) in the ALBI grade 1 group and 184 patients (48.7%) in the ALBI grade 2/3 group. In the whole cohort, 198 patients (52.4%) did not achieve TO, and the incidence of non-TO in the ALBI grade 2/3 group was obviously higher than that in the ALBI grade 1 group (*n* = 112, 60.9% *vs*. *n* = 86, 44.3%, *P* = 0.001). The multivariate analyses showed that ALBI grade 2/3 was an independent risk factor for non-TO (OR: 1.95, 95%CI: 1.30–2.94, *P* = 0.023).

**Conclusions:**

More than half (52.4%) of the patients with hepatocellular carcinoma did not achieve TO after laparoscopic hepatectomy, and preoperative ALBI grade 2/3 was significantly associated with non-TO. Improving the liver function reserve of patients before operation, thereby reducing the ALBI grade, may increase the probability for patients to reach TO and enable patients to benefit more from surgery.

## Introduction

Hepatocellular carcinoma (HCC) is the most common primary liver cancer, and it is expected that more than one million people will die from it in 2030 (1, 2). For resectable HCC patients, liver resection remains the preferred treatment with maximum benefit (3, 4). More and more minimally invasive procedures, especially laparoscopic, are being performed today, and their number has continued to grow exponentially (5–7).

The quality of surgery dramatically affects the short- and long-term prognosis of patients. Therefore, the evaluation of surgical quality is critical in clinical practice. In recent years, as a composite indicator, the effectiveness of “textbook outcomes (TO)” in evaluating surgical quality has been recognized by more and more scholars. TO can evaluate the overall quality of surgery more comprehensively than some other single-evaluation indexes, and it was a feasible and useful parameter for comparing the quality of institutions (8) as well as the assessment of patient-level outcomes, center designation, hospital performance, and quality metrics (9). At present, TO has been used to evaluate the quality of various types of surgery, and these studies have indicated that TO is associated with improved long-term outcomes after surgery (10–13).

Among many factors affecting the prognosis of HCC patients after hepatectomy, insufficient liver function reserve was considered as one of the most important risk factors (14). In 2015, Johnson et al. developed a new model for evaluating liver function, the albumin–bilirubin (ALBI) score (15). Once released, the model has been recognized by doctors for its good predictive value. Previous studies have shown that preoperative ALBI grade can affect the incidence of postoperative morbidity in patients with HCC, including liver failure, and a higher ALBI grade is an independent risk factor for prolonged hospital stay (16, 17). Therefore, in this study, we tended to study the association between preoperative ALBI grades and the incidence of TO or non-TO in patients with HCC undergoing laparoscopic hepatectomy.

## Methods

### Data source and patient selection

Patients with HCC who underwent laparoscopic hepatectomy in Zhejiang Provincial People’s Hospital from January 2015 to December 2020 were selected. The exclusion criteria include (1) recurrent hepatocellular carcinoma (2), younger than 18 years old when diagnosed (3), conversion to open surgery, and (4) missing important data. According to the preoperative ALBI score [(log_10_ bilirubin * 0.66) + (albumin * -0.085)], all patients were stratified as follows: ALBI grade 1 (ALBI ≤ -2.60) and ALBI grade 2/3 (ALBI > -2.60) (15). Patients with HCC were recognized by dynamic CT or MRI. If the imaging profile on CT or MR is specific for HCC (intense contrast uptake in the arterial phase followed by extracellular contrast wash-out in the venous and/or delayed phases), the diagnosis is established. Subsequently, the staging and treatment decisions for HCC are based on the Barcelona Clinic Liver Cancer staging standards. All patients with HCC were eventually diagnosed by the pathology of surgical specimens. This retrospective study complies with the Declaration of Helsinki and was approved by the institutional ethical committee, and the need for informed consent was waived.

### Clinical characteristics and operative variables

The study variables were retrospectively collected from the medical record system of Zhejiang Provincial People’s Hospital, including sex; age at diagnosis; body mass index (BMI); history of alcohol drinking, diabetes mellitus, and cigarette smoking; family history of HCC; performance status score; positivity of serum hepatitis B virus (HBV); presence of cirrhosis and portal hypertension; preoperative levels of alpha-fetoprotein (AFP); and platelet count. Tumor-related variables included tumor location, maximum diameter of tumor, tumor number, integrity of the tumor capsule, and macroscopic vascular invasion. Surgical variables included intraoperative blood loss, operation time, type of resection, and extent of hepatectomy (major or minor). According to the Brisbane 2000 nomenclature of liver anatomy and resections, the type of resection was divided into anatomical and non-anatomical resection (18). All serological samples were taken in the morning when the patient had fasted for more than 8, and the data were obtained before any treatment and less than 1 week before surgery. All serological test variables were uniformly tested by the clinic lab of our hospital.

### Definition of textbook outcome

TO is a composite indicator, which is composed of multiple indicators reflecting the short-term prognosis after surgery. We define it as follows (1): no morbidity within 30 days after surgery (2), no prolonged length of postoperative hospital stay (3), no perioperative blood transfusion (4), no readmission within 30 days after discharge (5), no mortality within 90 days after surgery, and (6) R0 resection. R0 resection was defined as microscopic resection margin that was negative (19). Postoperative morbidities included acute liver failure, hemorrhage, bile leakage, subdiaphragmatic effusion and abscess, infection in surgical incision, pulmonary infection, postoperative pleural effusion, postoperative subretinal effusion, and other complications. Prolonged length of postoperative hospital stay was defined as inpatient hospital stay that is longer than the 75th percentile of the postoperative length of stay (9 days). When a patient met the abovementioned six requirements, we determined that he reached TO. As such, non-TO was defined as follows: (1) morbidity occurred 30 days after operation, (2) prolonged length of postoperative stay, (3) perioperative blood transfusion, (4) readmission within 30 days after discharge, (5) postoperative 90-day mortality, and (6) R1 or R2 resection. R1 was defined as the presence of HCC upon microscopic observation. R2 was defined as the presence of HCC upon macroscopic observation. Meeting any of the points above is considered as non-TO.

### Statistical analysis

Data analysis was performed using IBM SPSS, version 25.0 (SPSS Inc.). Categorical variables were compared using *χ* ^2^ test with the Yates correction or the Fisher’s exact test as appropriate. Mann–Whitney *U*-test was used to compare the continuous variables. The baseline data of patients between ALBI grade 1 group and ALBI grade 2/3 group were compared. Significant variables (*P* < 0.1) in the univariable analysis were used to generate a multivariable logistic regression model. *P <*0.05 was set as a statistically significant difference.

## Results

### Baseline characteristics

**Figure 1:**
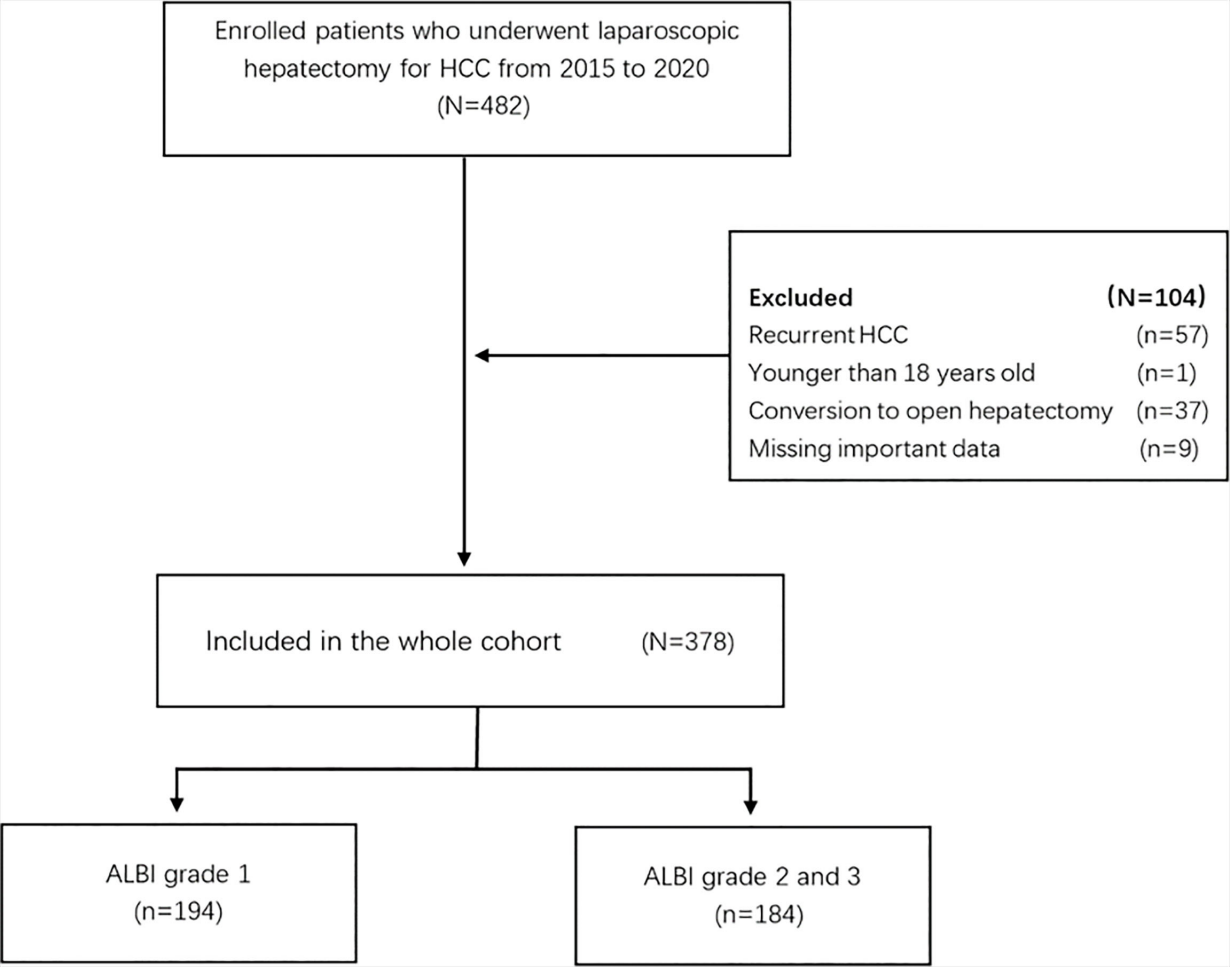
Flow chart of participant population. HCC, hepatocellular carcinoma; ALBI, albumin–bilirubin.

A total of 378 patients were enrolled in the whole cohort, including 194 cases (51.3%) in the ALBI grade 1 group and 184 cases (48.7%) in the ALBI grade 2/3 group (**Figure 1**). In the whole cohort, there was a total of 324 males (85.7%), more than half of whom had a history of chronic cirrhosis (*n* = 245, 64.8%), nearly a quarter of whom had portal hypertension (*n* = 95, 25.1%), only a minority of whom had family history of HCC (*n* = 39, 10.3%), and a minority of whom received major hepatectomy (*n* = 46, 12.2%). We can see that there is no significant difference in all clinical characteristics and surgically related variables between the ALBI grade 1 group and the ALBI grade 2/3 group (all *p >*0.05) (**Table 1**).

**Table 1:** Comparison of the clinical characteristics among the two groups according to preoperative albumin–bilirubin.

Variables	Overall (*N* = 378)	ALBI grade 1 (*n* = 194)	ALBI grade 2/3 (*n* = 184)	*P*
Male	324 (85.7%)	164 (84.5%)	160 (87.0%)	0.501
Age >60 years	155 (41.0%)	71 (36.6%)	84 (45.7%)	0.074
BMI^a^	22.9 (21.1–25.3)	23.1 (21.3–25.6)	22.7 (20.8–25.0)	0.164
Alcohol drinking	108 (28.6%)	50 (25.8%)	58 (31.5%)	0.216
Diabetes mellitus	48 (12.7%)	19 (9.8%)	29 (15.8%)	0.082
Cigarette smoking	153 (40.5%)	76 (39.2%)	77 (41.8%)	0.597
Family history of HCC	39 (10.3%)	21 (10.8%)	18 (9.8%)	0.739
Performance status ≥1	76 (20.1%)	32 (16.5%)	44 (23.9%)	0.072
HBV (+)	308 (81.5%)	159 (82.0%)	149 (81.0%)	0.806
Cirrhosis	245 (64.8%)	118 (60.8%)	127 (69.0%)	0.095
Portal hypertension	95 (25.1%)	41 (21.1%)	54 (29.3%)	0.066
AFP >400 ug/L	85 (22.5%)	42 (21.6%)	43 (23.4%)	0.689
PLT <100 μg/L	86 (22.8%)	37 (19.1%)	49 (26.6%)	0.080
Tumor in segment 7/8	115 (30.4%)	60 (30.9%)	55 (29.9%)	0.827
Maximum tumor size >2 cm	282 (74.6%)	137 (70.6%)	145 (78.8%)	0.068
Multiple tumors	56 (14.8%)	27 (13.9%)	29 (15.8%)	0.614
Incomplete capsule	248 (65.6%)	119 (61.3%)	129 (70.1%)	0.073
Microscopic vascular invasion	161 (42.6%)	82 (42.3%)	79 (42.9%)	0.896
Intraoperative blood loss >400 ml	81 (21.4%)	35 (18.0%)	46 (25.0%)	0.099
Operation time^a^	187.5 (140–250)	182.5 (130.0–240.0)	190.0 (150–258.8)	0.158
Non-anatomical resection	185 (48.9%)	97 (50.0%)	88 (47.8%)	0.673
Major hepatectomy	46 (12.2%)	20 (10.3%)	26 (14.1%)	0.256
TO/non-TO	180 (47.6%)/198 (52.4%)	108 (55.7%)/86 (44.3%)	72 (39.1%)/112 (60.9%)	0.001

a Values are median (range).

BMI, body mass index; HCC, hepatocellular carcinoma; HBV, hepatitis B virus; AFP, alpha-fetoprotein; PLT, platelet count; TO, textbook outcomes.

### Comparisons of TO and non-TO

In the whole cohort, a total of 198 cases (52.4%) did not achieve TO. There were 127 patients (33.6%) who experienced morbidity within 30 days after surgery, and 85 (22.5%) patients had a prolonged length of postoperative hospital stay. The median hospital stay was 8 (range, 6–9) days, and 96 (25.4%) patients received perioperative blood transfusion. Only a small number of patients experienced readmission within 30 days (*n* = 6, 1.6%), mortality within 90 days after surgery (*n* = 3, 0.8%), and R1 and R2 resection (*n* = 3, 0.8%).

**Figure 2:**
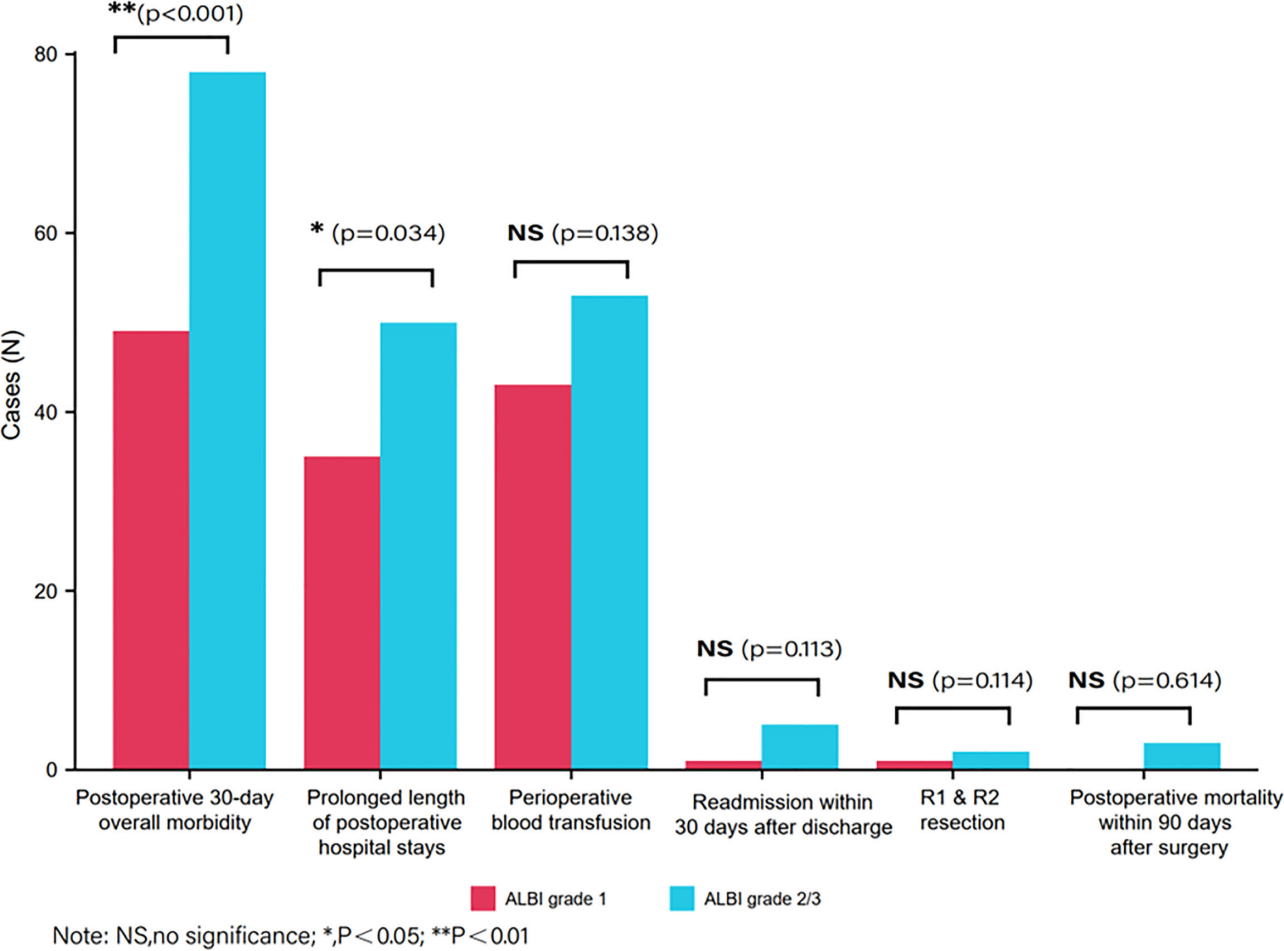
Distribution of achieving a non-textbook outcome between two groups according to preoperative albumin–bilirubin.

**Figure 2** describes the distribution of achieving a non-textbook outcome among two groups according to preoperative ALBI. As we can see in **Table 2**, the incidence of non-TO in the ALBI grade 2/3 group was significantly higher than that in the ALBI grade 1 group (*n* = 112, 60.9% *vs*. *n* = 86, 44.3%, *p* = 0.001). By comparing the distribution of achieving a non-TO, we can see that the number of patients who experienced morbidity within 30 days after surgery in the ALBI grade 2/3 group was significantly higher than that in the ALBI grade 1 group (*n* = 78, 42.4% *vs*. *n* = 49, 25.3%, *p* < 0.001). There were 50 (27.2%) patients with prolonged postoperative hospital stay in the ALBI grade 2/3 group, and the median postoperative hospital stay was 8 (7–10) days, while the ALBI grade 1 group had 35 (18%) patients, and the median hospital stay was 7 (7–9) days (*P* < 0.001). In the other four individual outcome parameters, there was no significant difference between the two groups (*P* > 0.05).

**Table 2:** Distribution of achieving non-textbook outcomes.

Distribution of non-textbook outcomes	Overall (*N* = 378)	ALBI grade 1 (*n* = 194)	ALBI grade 2/3 (*n* = 184)	P
Overall	198 (52.4%)	86 (44.3%)	112 (60.9%)	0.001
Morbidity within 30 days after surgery	127 (33.6%)	49 (25.3%)	78 (42.4%)	<0.001
Prolonged length of postoperative hospital stays^a^	85 (22.5%)	35 (18.0%)	50 (27.2%)	0.034
Postoperative hospital stays, days^b^	8 (6–9)	7 (7–9%)	8 (7–10%)	<0.001
Perioperative blood transfusion	96 (25.4%)	43 (22.2%)	53 (28.8%)	0.138
Readmission within 30 days after discharge	6 (1.6%)	1 (0.5%)	5 (2.7%)	0.113
Mortality within 90 days after surgery	3 (0.8%)	0	3 (1.6%)	0.114
R1 a**nd** R2 resection	3 (0.8%)	1 (0.5%)	2 (1.1%)	0.614

a Prolonged length of postoperative hospital stay was defined as an inpatient hospital stay longer than the 75th percentile of postoperative length of stay (9 days).

b Values are median (range).

### Independent risk factors associated with non-TO

**Table 3** shows the results of the regression analyses for patients with HCC who did not achieve TO after laparoscopic hepatectomy. According to the results, ALBI grade 2/3 was an independent risk factor for non-TO (OR: 1.95, 95%CI: 1.30–2.94, *P* = 0.023). In addition, the multivariate regression analysis also shows that BMI ≥25 (OR: 1.87, 95%CI: 1.10–3.18, *P* = 0.022), diabetes mellitus (OR: 2.80, 95%CI: 1.32–5.94, *P* = 0.007), portal hypertension (OR: 2.01, 95%CI: 1.15–3.51, *P* = 0.014), multiple tumor (OR: 2.07, 95%CI: 1.02–4.19, *P* = 0.043), intraoperative blood loss >400 ml (OR: 4.73, 95%CI: 2.38–9.40, *P* < 0.001), operation time ≥180 min (OR: 2.25, 95%CI: 1.38–3.66, *P* = 0.001), and major hepatectomy (OR: 3.22, 95%CI: 1.40–7.42, *P* = 0.006) were also associated with a higher incidence of non-TO.

**Table 3:** Univariable and multivariable logistic regression analyses of risk factors associated with non-TO following laparoscopic hepatectomy for HCC.

Variables	OR Comparison	UV OR (95%CI)	UV *P*	MV OR (95%CI)	MV *P* ^a^
Sex	Male *vs*. female	0.94 (0.53–1.68)	0.834		
Age	>60 *vs*. ≤60 years	1.04 (0.69–1.56)	0.865		
BMI	≥25 *vs*. <25 kg/m^2^	1.93 (1.21–3.08)	0.006	**1.87** (**1.10**–**3.18**)	**0.022**
Alcohol drinking	Yes *vs*. no	1.26 (0.80–1.97)	0.313		
Diabetes mellitus	Yes *vs*. no	3.11 (1.56–6.19)	0.001	**2.80** (**1.32**–**5.94**)	**0.007**
Cigarette smoking	Yes *vs*. no	1.13 (0.75–1.71)	0.549		
Family history of HCC	Yes *vs*. no	0.85 (0.44–1.65)	0.629		
Performance status	≥1 *vs*. <1	1.41 (0.85–2.35)	0.183		
HBV (+)	Yes *vs*. no	1.20 (0.72–2.03)	0.480		
Cirrhosis	Yes *vs*. no	1.65 (1.08–2.52)	0.022	NA	0.195
Portal hypertension	Yes *vs*. no	1.91 (1.18–3.09)	0.008	**2.01** (**1.15**–**3.51**)	**0.014**
ALBI	Grade 2/3 *vs*. grade 1	1.95 (1.30–2.94)	0.001	**1.73** (**1.08**–**2.77**)	**0.023**
AFP	>400 *vs*. ≤400 ug/L	1.32(0.81–2.14)	0.270		
PLT	<100 *vs*. >100 ug/L	1.35 (0.83–2.20)	0.225		
Tumor in segment 7/8	Yes *vs*. no	1.56 (1.00–2.43)	0.051	NA	0.379
Maximum tumor size	>2 *vs*. ≤2 cm	1.89 (1.18–3.02)	0.008	NA	0.420
Tumor number	Multiple *vs*. solitary	2.60 (1.40–4.82)	0.003	**2.07** (**1.02**–**4.19**)	**0.043**
Capsule	Incomplete *vs*. complete	1.21 (0.79–1.85)	0.375		
Microscopic vascular invasion	Yes *vs*. no	1.67 (1.10–2.52)	0.015	NA	0.659
Intraoperative blood loss	>400 *vs*. ≤400 ml	6.72 (3.56–12.70)	<0.001	**4.73** (**2.38**–**9.40**)	**<0.001**
Operation time	≥180 *vs*. <180 min	3.38 (2.20–5.16)	<0.001	**2.25** (**1.38**–**3.66**)	**0.001**
Type of resection	Non-anatomical *vs*. anatomical	0.88 (0.59–1.32)	0.550		
Extent of hepatectomy	Major *vs*. minor	4.37 (2.04–9.33)	<0.001	**3.22** (**1.40**–**7.42**)	**0.006**

a Those variables found to be significant at P <0.1 in the univariable analysis were entered into a multivariable logistic analysis.

BMI, body mass index; HCC, hepatocellular carcinoma; HBV, hepatitis B virus; AFP, alpha-fetoprotein; PLT, platelet count. MV, multivariable; NA, not available; OR, odds ratio; UV, univariable.

The bold values means that those variables found to be significant at P <0.05 in the multivariate analysis.

## Discussion

The quality of surgery is closely related to the short- and long-term prognosis, so it is very important to evaluate the quality of surgery effectively. Surgical medical quality assessment is multidimensional and multi-level. For the evaluation of surgical quality, some single-evaluation indexes were used in the past, such as R0 resection, intraoperative blood loss, perioperative mortality, postoperative morbidity, and hospitalization time (20, 21); however, this often cannot accurately and comprehensively evaluate the surgical quality from multiple levels. Therefore, as a composite index, TO combines multiple parameters into a single defined quality index, which can more accurately evaluate the overall quality of surgery. Since Kolfschoten first proposed the concept of TO in 2013 and applied it to evaluate the quality of colon cancer surgery (12), TO is also gradually being used to evaluate the quality of other surgeries, including hepatectomy, liver transplantation, and other liver-related surgeries (22, 23).

In the present study, we employed the novel use of the indicator of TO to comprehensively evaluate perioperative outcomes for patients with HCC after hepatectomy. The results showed that 180 (47.6%) of the patients reached TO in the cohort, which was higher than that in previous studies (33.3%) (24). Because the cohorts were all laparoscopic hepatectomy patients, excluding open surgery patients, and a previous meta-analysis showed that laparoscopic hepatectomy had a lower incidence of postoperative morbidity, shorter postoperative hospital stays, and less intraoperative blood loss than open hepatectomy (25–28), these factors may lead to higher TO rates in this cohort than that in other studies. It can be seen from the results of this study that the main influencing factors for non-TO were postoperative morbidity (33.6%), prolonged postoperative hospital stays (22.5%), and perioperative blood transfusion (25.4%), while there are very few patients with non-TO due to the other three factors.

Previous studies have shown that insufficient liver function reserve is considered one of the most important risk factors in various clinical and operative variables associated with postoperative morbidity (29–31). At present, Child–Pugh grading is widely used to evaluate liver function reserve; however, this indicator contains two subjective parameters (ascites and hepatic encephalopathy), which can lead to an inconsistent Child–Pugh classification due to the differences in subjective judgments among different observers. Johnson (15) established that the ALBI classification model only involves two simple objective indicators, which are convenient to calculate, and has been proved to be superior to the traditional Child–Pugh classification in evaluating liver function, postoperative liver failure, and prognosis of HCC (32, 33). A previous study demonstrated the relationship between BMI and TO in patients with HCC (34), but the association between liver function reserve and TO has not been reported. We can detect from the results of this study that ALBI grade 2/3 was an independent risk factor for non-TO [OR: 1.95 (1.30–2.94), *P* = 0.023]. Therefore, we may lower the preoperative AIBL grade of patients through preoperative albumin infusion and other interventions, thereby increasing the probability of TO and increasing the benefits of surgery.

According to the results of multivariate regression analyses, we found that, except ALBI grade 2/3, BMI ≥25, portal hypertension, blood loss >400 ml, and major liver resection were also independently associated with a higher incidence of non-TO, which was consistent with previous studies (34). In addition, this study showed that diabetes mellitus and operation time ≥180 min also play a negative effect on non-TO, which has not been reported before. The possible reason is that the immune function of diabetic patients was worse than that of normal people, and the probability of postoperative morbidity such as infection was relatively high, resulting in an increase in the probability of non-TO. In addition, the prolongation of operation time often indicated the increase of intraoperative bleeding, which may lead to the increase of perioperative blood transfusion rate. On the other hand, the increase of operation time also increases the possibility of postoperative morbidity, such as infection, which will increase the probability of non-TO. It is worth noting that this study suggested that cirrhosis is not an independent risk factor of non-TO, which contradicted previous studies (34). The reason may be the following: first, the cohort of patients were resectable HCC patients, and the majority of patients in the cohort had cirrhosis in the compensatory phase; second, the vast majority of patients with cirrhosis in this cohort were cases of hepatitis B-related cirrhosis, most patients were regularly treated with antiviral therapy before surgery, the process of cirrhosis was effectively controlled, and only a small number (12.2%) of patients in this cohort received major hepatectomy, so the risk of postoperative liver failure was small. The abovementioned reasons may lead to cirrhosis not becoming an independent risk factor for non-TO.

There are several undeniable limitations in this study. First, this is a single-center, retrospective study; the retrospective design introduces the risk of selection bias. Second, the majority of the included patients were HBV-related HCC, and whether the same results exist in western countries (mainly HCV-related HCC) remains to be studied. Third, this study only enrolled patients undergoing laparoscopic hepatectomy. Compared with open hepatectomy, laparoscopic hepatectomy had a lower incidence of postoperative morbidities, less intraoperative blood loss, and shorter postoperative hospital stays (25–28). These factors can directly affect the probability of patients reaching TO. Fourth, the definition of TO, however, is arbitrary, subject to cultural differences, and might even change over time (35). Therefore, the definition of TO in this study was not necessarily applicable to other regions, which may also lead to differences in results.

In conclusion, more than half (52.4%) of patients with hepatocellular carcinoma did not achieve TO after laparoscopic hepatectomy, and preoperative ALBI grade 2/3 was independently associated with non-TO for HCC flowing laparoscopic hepatectomy. Improving the liver function reserve of patients before operation, thereby reducing the ALBI grade, may increase the probability for patients to reach TO and enable the patients to benefit more from surgery.

## Data availability statement

The raw data supporting the conclusions of this article will be made available by the authors without undue reservation.

## Ethics statement

This retrospective study complies with the Declaration of Helsinki and was approved by the institutional ethical committee and the need for informed consent was waived.

## Author contributions

F-QX, T-WY, D-DW, and Y-MX contributed equally to this work. LL and Dong-Sheng Huang had full access to all the data in the study and takes responsibility for the integrity of the data and the accuracy of the data analysis. Study concept and design: F-QX, LL, and D-SH. Acquisition, analysis, or interpretation of data: T-WY, D-DW, Y-MX, K-JZ, JC, Z-QX, S-YL, W-FY, and KJ. Drafting of the manuscript: F-QX and T-WY. Critical revision of the manuscript for important intellectual content: G-LS, J-WL, and C-WZ. Statistical analysis: T-WY, D-DW, and Y-MX. Obtained funding: LL, W-FY, and KJ. Administrative, technical, or material support: C-WZ, D-SH, and LL. Study supervision: D-SH and LL. All authors listed have made a substantial, direct, and intellectual contribution to the work and approved it for publication.

## Funding

Funding for this study was provided by Zhejiang Provincial People’s Hospital (no. ZRY2020A004), Health Commission of Zhejiang Province (no. 2022KY532, no. 2018KY261, and no.2018277275), and General Scientific Research Project of the Education Department of Zhejiang Province (no. Y201840617). The funding sources had no role in the design and conduct of the study; collection, management, analysis, and interpretation of the data; preparation, review, or approval of the manuscript; and decision to submit the manuscript for publication.

## Conflict of interest

The authors declare that the research was conducted in the absence of any commercial or financial relationships that could be construed as a potential conflict of interest.

## Publisher’s note

All claims expressed in this article are solely those of the authors and do not necessarily represent those of their affiliated organizations, or those of the publisher, the editors and the reviewers. Any product that may be evaluated in this article, or claim that may be made by its manufacturer, is not guaranteed or endorsed by the publisher.

## Abbreviations

HCC, hepatocellular carcinoma; ALBI, albumin–bilirubin; TO, textbook outcomes; BMI, body mass index; PS, performance status; PLT, platelet count; HBV, hepatitis B virus; AFP, alpha-fetoprotein; MV, multivariable; NA, not available; OR, odds ratio; UV, univariable; CI, confidence interval.
